# Career transition plans of veterinarians in clinical practice

**DOI:** 10.3389/fvets.2024.1433891

**Published:** 2024-07-26

**Authors:** Lori R. Kogan, Mark Rishniw

**Affiliations:** ^1^College of Veterinary Medicine and Biomedical Sciences, Colorado State University, Fort Collins, CO, United States; ^2^Veterinary Information Network, Davis, CA, United States

**Keywords:** retirement, burnout, job fulfillment, workplace stressors, career

## Abstract

**Objective:**

Gain an understanding of the career transition plans of veterinarians in clinical practice.

**Sample:**

Veterinary members of the Veterinary Information Network (VIN) working as small animal clinicians.

**Procedures:**

An electronic survey distributed via the VIN data collection portal.

**Results:**

A total of 1,256 responses from veterinarians in clinical practice were analyzed, with 61% indicating they plan to decrease their clinical work, and 31% to stop entirely within the next 5 years. The most common reasons for these choices were to have more free time for oneself and/or family/friends (76%), to maintain good health (59%), and feeling burned out (50%). Factors that might entice them to retain their current number of clinical hours included reduced workload or shorter hours (42%), financial incentivization (38%), and improved working conditions (26%). Concerns related to retirement were common with 47% of participants in our study reported feeling concerned about the loss of professional identity, 34% reported concern about reduced social connections, and 28% reported concern as to how they would fill their time.

**Conclusions and clinical relevance:**

The reported desire to reduce/stop one’s clinical work within the next 5 years by 42% of veterinarians ≤44 years of age, with burnout a primary predictor, offers insights into the necessity of change at the organizational, systemic (versus individual) level. The fact that many participants reported concerns related to retirement and 32% reported that they did not have adequate retirement information suggests a need for supportive services to help ensure a successful transition.

## Introduction

The current average retirement age for workers in the US is 65 years for men and 62 years for women (1). In 2022, the average age of male veterinarians in the workplace was 50.5 and 41.3 for female veterinarians (2). It is therefore likely that many veterinarians will retire over the two decades. These demographics, as well as the fact that the mean age of graduation has been steadily increasing since 1975 (thereby potentially creating shorter career lengths) has the potential to reduce the number of practicing veterinarians (3).

In addition to retirement, approximately 30% of companion animal veterinarians have indicated they would like to work fewer hours, with top cited reasons involving work-life balance and mental health (e.g., stress, anxiety, and burnout) (4). Veterinarians are at a particularly high risk for burnout due to a myriad of challenges including long work hours, on-call duties, moral distress, ethical dilemmas, poor work-life balance, client expectations, lack of managerial support, and understaffing (5–9). Work-related stressors and burnout also contribute to the decision to retire (10–13).

Other factors that may influence veterinarians’ decisions to reduce their clinical practice hours or retire early include physical and cognitive changes (10, 14). Healthy human brains undergo significant changes with age, often beginning before 60 years old, with reductions in processing speed, executive functioning, and episodic memory (15). To our knowledge, no studies on aging veterinarians’ cognitive and physical changes have been conducted, yet several studies have focused on the need to help support aging physicians experiencing cognitive and/or physical decline (16, 17).

The decision to retire or reduce one’s clinical workload is often difficult and some professionals struggle with potential negative impacts pertaining to finances, a loss of identity, and a reduction in social interactions (18, 19). Despite high levels of burnout, and possible physical or cognitive decline, veterinarians might elect to continue working because of financial concerns. When compared to similar professions, veterinarians make lower wages, yet have similar amounts of debt. For example, the average physician’s annual salary is $239,000 compared to $147,787 for the average veterinarian in all types of veterinary-related work, despite having similar student debt (20, 21). The educational debt of veterinary student graduates averaged $154,451 in 2023 ($185,486 for those with at least some loans), with 33% of veterinary students graduating with a debt of $200,000 or more (20). Even though debt load has declined since 2020, it remains a concern (20, 22). Given that it takes 20 years, on average, for students to repay their student debt (23), many veterinarians might be unable to retire when they want due to financial limitations. To our knowledge, there have been no studies examining the financial factors that impact veterinarians’ decision to retire, however, several studies have found that physicians’ retirement decisions are impacted by finances (19, 24, 25). In addition to financial concerns, veterinarians may be worried about a loss of identity. Similar to physicians, many veterinarians’ identification as a doctor is central to their person (18, 26), creating challenges when making retirement decisions (19, 27, 28).

Despite the growing number of aging veterinarians, little is known about practicing veterinarians’ plans to reduce their clinical hours or retire. Therefore, we sought to better understand the reasons why practicing veterinarians are contemplating a reduction in clinical hours, or retirement, as well as potential incentives that might make them reconsider. We also wanted to assess any perceived changes of cognitive and physical skills, within the past 5 years, related to clinical work. Lastly, for those contemplating retirement, we wanted to better understand their concerns, and where they access their retirement information to determine any unmet retirement-centered needs.

## Materials and methods

We created an online survey and distributed a link to the survey via email to members of the Veterinary Information Network, an online community for veterinarians (*n* ~ 43,000). Participants could anonymously access the survey from March 1, 2024 to March 24, 2024. A follow-up message was sent 2 weeks after the initial invitation. We included data only from respondents who stated they currently worked as veterinarians in clinical practice but excluded those practicing as interns or residents. The study was categorized as exempt by Colorado State University’s Institutional Review Board. The survey was administered directly via the VIN data collection portal, and branching logic was used to display only relevant questions to each participant.

The body of the survey consisted of demographic questions including age, gender, relationship status (partnered/married or single), number of children under 18 for whom they are responsible, if they are responsible for the care of elderly/disabled family members (yes/no), and if they have any outstanding large debts (yes/no). Participants were asked to indicate the proportion of their clinical work spent with small animals, large animals, horses, exotic animals, and wildlife. They were also asked to describe their work setting (brick-and-mortar private practice, brick-and-mortar corporate practice, mobile, relief, academic, shelter).

Participants were asked to indicate the statement that best reflects their current work/life balance using a 5-point Likert scale with 1 = I feel I work way too much, leaving little to no time for home/personal life and 5 = I feel I would like to work a great deal more, I have too much time for home/personal life. Using an 11-point scale, with 0 = very unsatisfied and 10 = very satisfied, they were asked to rate their current level of job satisfaction with their clinical veterinary work.

Burnout and professional fulfillment were assessed with the Stanford Professional Fulfillment Index (PFI) (29). The PFI is a 16-item survey with three scales: two scales measure burnout in terms of work exhaustion (four questions) and interpersonal disengagement (six questions); and one scale that measures professional fulfillment (six questions). Response options are on a f5-point Likert scale (“not at all true” to “completely true”) for professional fulfillment items and “not at all” to “extremely” for work exhaustion and interpersonal disengagement items. Items are scored 0–4 with each dimension treated as an integer variable. Scale scores are calculated by averaging the item scores of all the items within the corresponding scale. Higher scores on the professional fulfillment scale are viewed more favorably while higher scores on the work exhaustion or interpersonal disengagement scales are less favorable. Dichotomous burnout categories are determined from the average item score of all 10 burnout items (work exhaustion and interpersonal disengagement), using a cut-point of 1.33. Dichotomous professional fulfillment is recommended at an average item score cut-point of ≥3.0 (29).

Participants were asked if they plan to change the amount or type of paid work they do within the next 5 years. For those who indicated they plan a change, they were asked how they plan to change their clinical veterinary work (increase, leave unchanged, decrease, stop entirely). The participants who indicated they plan to decrease or stop their clinical veterinary work were asked to indicate, from a list of 16 factors, those that they feel are reasons for the desired change. Examples of reasons include “Feeling burned out,” “To have more free time for self ± family/friends,” and “personal health problems.” They were then asked to assign a level of importance to each selected reason using a 5-point scale (1 = not very important to 5 = exceptionally important). They were then asked to indicate from a list of eight factors, those factors that might entice them to retain their current number of clinical hours (e.g., “Reduction of on-call or emergency commitments” and “Development opportunities”).

Participants were next asked to compare their current status with that of 5 years ago (greater, about the same, somewhat less, considerably less) on several job-related factors. Examples include “ability to manage a heavy patient load,” “ability to practice veterinary medicine the way you want based on your physical health” and “ability to incorporate new modalities of diagnosis and treatment into your practice.”

Participants were then asked at what age they visualize transitioning away entirely (either to another type of work or retirement) from clinical veterinary work. Participants 50 and older were asked their agreement level, using a 5-point Likert scale, with potential concerns about retirement (e.g., loss of professional identity, reduced social connections). They were also asked where they go for retirement information/guidance (e.g., personal advisor, AVMA or national veterinary association, AARP or national aging association, family/friends). Lastly, they were asked if they feel they have sufficient information about retirement (yes/no).

Descriptive statistics, Chi Square, Analysis of Variance, and binary regression were performed with SPSS, version 28. To determine if personal demographics could predict estimated age for retirement, logistic regression was used to predict estimated age for retirement based on participants’ gender, relationship status, children status, type of employment, debt, job fulfillment, and burnout. Significance level was set at 0.05.

## Results

### Demographics

We received 1,256 responses from veterinarians in clinical practice, of which 920/1,249 (73.7%) were females, and 329/1,249 (26.3%) were males. Most (973/1,252, 77.7%) were partnered/married, did not have any children under 18 years of age (947/1,256, 75.4%), were not responsible for the care of an elderly or disabled family member (1,035/1,252, 82.7%), but more than half had outstanding large debts (742/1,255, 59.1%). Most (1,153/1,250, 92.2%) reported working with small animals 90% or more of the time, with most working in a brick-and-mortar practice [either private (50.9%) or corporate (32.5%)]. Of the remaining respondents, 43/1,203 (3.6%) worked as mobile veterinarians, 96/1,203 (8.0%) as relief veterinarians, 34/1,203 (2.8%) as academic clinicians, and 27/1,203 (2.2%) as shelter veterinarians.

### Wellbeing

Participants rated their current level of job satisfaction with their clinical work using an 11-point scale (0 very low =10 very high) (*n* = 1,248), with an average rating of 6.75 (SD = 2.30). Age affected clinical job satisfaction (*p* < 0.001), with participants ≤44 years of age reporting lower satisfaction (*M* = 5.86, SD = 2.23) than older participants (*M* = 6.98, SD = 2.26). Participants ≥65 year of age reported higher satisfaction than younger participants (*M* = 7.79, SD = 2.03). Males reported higher satisfaction (*M* = 7.18, SD 2.31) than females (*M* = 6.60, SD = 2.28) (*p* < 0.001). When asked about their current work/life balance (*n* = 1,187), the largest number (565, 45.3%) reported feeling they work somewhat too much, followed by 481 (38.3%) who reported feeling they work the right amount. Relatively few participants reported working way too much (175, 13.9%), and very few would like to work more (27, 2.2%). Males were more likely to report working the right amount (159/345, 46.1%) compared to females (353/966, 36.5%) (*p* = 0.039). Participants ≥65 year of age were more likely to report feeling they work the right amount (123/253, 48.6%) (*p* = 0.013) compared to younger participants (≤44 years of age: 90/269, 33.5%; 45–54 years of age: 110/324, 34.0%; 55–64 years of age: 190/470, 40.4%).

Job burnout was assessed by averaging the 10 items related to burnout (work exhaustion and interpersonal disengagement) on the Stanford Professional Fulfillment Index (PFI) (29). Using the suggested dichotomous burnout cut off score of 1.33, a total of 426/1,252 (34.0%) of respondents fell above the cutoff for burnout. There were no differences based on gender (*p* = 0.274), relationship status (*p* = 0.195), children status (*p* = 0.442), or responsibility for elderly/disabled family member (*p* = 0.087). Younger participants, however, had burnout scores more frequently than older participants (*p* < 0.001, *R*^2^ = 0.070).

Job fulfillment was assessed by averaging the six questions of the PFI, using the cut off score of >3.0. The number of participants who scored higher than the cut off score was 503/1,252 (40.2%). There were no differences based on gender (*p* = 0.373), relationship status (*p* = 0.072), or parent status (*p* = 0.312). However, participants responsible for an elderly/disabled family reported lower fulfillment (*p* = 0.010, *R*^2^ = 0.003), as did younger participants (*p* < 0.001, *R*^2^ = 0.075).

### Future career plans

Participants were asked if, and how, they plan to change their amount of clinical work over the next 5 years. The majority (547/898, 60.9%) reported they plan to decrease their clinical work, 275/898 (30.6%) reported they plan to stop entirely, 41/898 (4.6%) reported planning no change, and 35/898 (3.9%) reported a plan to increase clinical work. When analyzed by age brackets, older participants were more likely to report the intention to stop or decease their clinical veterinary work (*p* < 0.001) (Table 1).

**Table 1:** Participants’ plans to change the amount of clinical work they perform over the next 5 years by age.

	Stop entirely		Decrease	Leave unchanged	Increase	Total
44 and younger	*N*	20	93	137	19	269
	%	7.4%	34.6%	50.9%	7.1%	100.0%
45–54	*N*	41	121	147	15	324
	%	12.7%	37.3%	45.4%	4.6%	100.0%
55–64	*N*	123	223	105	7	458
	%	26.9%	48.7%	22.9%	1.5%	100.0%
65 and older	*N*	96	112	40	2	250
	%	38.4%	44.8%	16.0%	0.8%	100.0%

Binary regression was conducted to determine predictors of planned changes (stop/reduce and maintain/increase) in the amount of their clinical work over the next 5 years. The potential predictors included burnout, fulfillment, children (yes/no), gender (male/female), relationship status (partnered/single), responsible for care of elderly or disabled family members (yes/no), outstanding large debt (yes/no) and age (44 and younger, 45–54, 55–64, 65 and older). Significant predictors included burnout (higher levels of burnout more likely to stop/reduce; *B* = −0.501; CI: 0.489–0.750; *p* < 0.001), fulfillment (higher levels of fulfillment less likely to stop/reduce; *B* = 0.333; CI: 1.155–1.686; p < 0.001), children (those with children less likely to stop/reduce; *B* = 0.365; CI: 1.044–1.988; *p* = 0.026), relationship status (single less likely to stop/reduce; *B* = 0.450; CI: 1.134–1.518; *p* = 0.006), outstanding debt (those with debt less likely to stop/reduce; *B* = 0.493; CI: 1.230–2.180; *p* < 0.001) and age [44 and younger years of age (*p* = 0.002)], 45–54 years of age (*p* < 0.001), and 55–64 years of age (*p* > 0.001) were all less likely to report they plan to stop/decrease their clinical work when compared to participants 65 years and older (Table 2).

**Table 2:** Results of binary regression predicting plans to stop/decrease or remain same/increase clinical work within next 5 years based on burnout, fulfillment, child status, gender, relationship status, responsible for care of elderly or disabled family members, outstanding large debt, and age.

	*B*	S.E.	Wald	df	Sig.	Exp (*B*)	95% C.I. for Exp (*B*)	
							Lower	Upper
Burnout	−0.501	0.109	21.128	1	<0.001	0.606	0.489	0.750
Fulfillment	0.333	0.097	11.923	1	<0.001	1.396	1.155	1.686
Children	0.365	0.164	4.928	1	0.026	1.441	1.044	1.988
Gender	0.100	0.162	0.382	1	0.537	1.105	0.805	1.518
Relationship status	0.450	0.165	7.410	1	0.006	1.569	1.134	2.170
Care for elderly family	0.153	0.181	0.714	1	0.398	1.165	0.818	1.660
Outstanding large debts	0.493	0.146	11.411	1	<0.001	1.638	1.230	2.180
Age			112.164	3	<0.001			
Age 45–54	−0.581	0.187	9.674	1	0.002	0.559	0.388	0.807
Age 55–64	−1.736	0.201	74.945	1	<0.001	0.176	0.119	0.261
Age 65 and older	−2.381	0.260	83.965	1	<0.001	0.092	0.056	0.154

Those who reported they plan to decrease or stop their clinical work (*n* = 822) were asked to indicate, from a list of 16 factors, the reasons for the desired change. The most commonly cited reasons were “to have more free time for self ± family/friends” (628, 76.4%), “to maintain good health” (482, 58.6%) and “feeling burned out” (414, 50.4%) (Table 3).

**Table 3:** Reasons given by veterinarians in clinical practice to decease or stop clinical work within the next 5 years (*n* = 822).

	*N* ^*^	Percent
To have more free time for self ± family/friends	628	76.4%
To maintain good health	482	58.6%
Feeling burned out	414	50.4%
Reduced job satisfaction	287	34.9%
Pressure of work	247	30.0%
Possibility of deteriorating physical ability needed to perform clinical work	207	25.2%
Financial security/insufficient financial incentive to stay	189	23.0%
Retirement of spouse/partner	155	18.9%
To pursue non-veterinary business or employment ventures	141	17.2%
Work schedule (overnights, on call, etc.)	117	14.2%
Personal health problems	115	14.0%
Family members’ health problems	96	11.7%
Possibility of deteriorating cognitive skills needed to perform clinical work	80	9.7%
Declining reimbursement for clinical care	58	7.1%
To pursue veterinary-related administrative/leadership opportunities	47	5.7%
To pursue veterinary-related research or medical education opportunities	36	4.4%

* Could select more than one response.

Participants were then asked to indicate from a list of eight factors, the factors that might entice them to retain their current number of clinical hours (*n* = 822). The factors identified as enticing by the largest number of respondents included a reduced workload or shorter hours per shift (348, 42.3%), financial incentivization (311, 37.8%), and improved working conditions other than hours (215, 26.2%) (Table 4).

**Table 4:** Possible incentives to make veterinarians in clinical practice retain their current number of clinical hours (*n* = 822).

	*N* ^*^	Percent
Work-load reduction/shorter hours	348	42.3%
Financial incentivization	311	37.8%
Reduction of work-related bureaucracy	212	25.8%
Development opportunities	89	10.8%
Less involvement in direct patient care	82	10.0%
Reduction of on-call or emergency commitments	71	8.6%
More involvement in direct patient care	23	2.8%

* Could select more than one response.

Participants 50 years of age and older were next asked to compare their current status with that of 5 years ago (greater, about the same, somewhat less, considerably less, not applicable) on several job-related factors. The factors with the greatest number of participants noting a decline included ability to recover from a night shift, (182/241, 75.5%), ability to recover from a long shift (528/822, 64.3%), and ability to deal with difficult personalities at work (372/849, 43.9%) (Table 5). Reported changes based on age brackets (50–54, 55–64, and 65 and older) are reported in Table 6. Significant differences were found for ability to manage a heavy patient load (*p* = 0.005), ability to perform common procedures (*p* = 0.006), ability to manage complicated clinical problems (*p* = 0.016), handle the stresses of veterinary medicine (*p* < 0.001), empathize with clients (*p* < 0.001), and level of emotional exhaustion (*p* = 0.023). In each of these, older participants, when compared to younger participants, were either more likely to report their current status was less than it was 5 years ago or less likely to report their ability was greater than it was 5 years ago. Participants were also asked to rate their current physical health (*n* = 915). The majority rated their health as good (221, 24.2%), very good (349, 38.1%) or excellent (216, 23.6%). There was no difference in health rating based on age.

**Table 5:** Reported change by participants 50 years of age and older in current status compared to 5 years ago for 12 factors related to clinical practice.

	Greater		About the same		Somewhat less		Considerably less	
Ability to recover from a night shift	12	5.0%	47	19.5%	59	24.5%	123	51.0%
Ability to recover from a long shift	54	6.6%	240	29.2%	327	39.8%	201	24.5%
Ability to deal with difficult personalities at work	143	16.8%	334	39.3%	223	26.3%	149	17.6%
Ability to handle the stresses of veterinary medicine	102	11.9%	353	41.0%	281	32.7%	124	14.4%
Ability to manage a heavy patient load	41	4.8%	357	41.9%	341	40.0%	114	13.4%
Ability to practice veterinary medicine the way you want based on your physical health	38	4.4%	470	54.9%	269	31.4%	79	9.2%
Ability to perform common procedures (e.g., physical exams, surgery, handling animals)	37	4.3%	554	64.8%	218	25.5%	46	5.4%
Ability to empathize with clients	138	16.1%	535	62.5%	138	16.1%	45	5.3%
Ability to incorporate new modalities of diagnosis and treatment into your practice	104	12.2%	551	64.5%	173	20.3%	26	3.0%
Your memory	16	1.9%	538	62.7%	284	33.1%	20	2.3%
Ability to manage complicated clinical problems	83	9.7%	594	69.6%	167	19.6%	10	1.2%
Ability to practice veterinary medicine the way you want based on your cognitive abilities	44	5.2%	654	76.9%	144	16.9%	9	1.1%

**Table 6:** Reported change by participants 50–54, 55–64, and 65 and older years of age in current status compared to 5 years ago for 12 factors related to clinical practice.

		50–54		55–64		65 and older	
		*N*	%	*N*	%	*N*	%
Your ability to practice veterinary medicine the way you want based on your physical health (*p* = 0.089)	Greater	15	8.6%	13	3.0%	10	4.1%
	About the same	91	52.3%	247	56.3%	131	54.1%
	Somewhat less	54	31.0%	141	32.1%	74	30.6%
	Considerably less	14	8.0%	38	8.7%	27	11.2%
Your ability to practice veterinary medicine the way you want based on your cognitive abilities (*p* = 0.755)	Greater	12	6.9%	17	3.9%	15	6.3%
	About the same	133	76.0%	341	78.0%	179	75.2%
	Somewhat less	28	16.0%	75	17.2%	41	17.2%
	Considerably less	2	1.1%	4	0.9%	3	1.3%
Your ability to manage a heavy patient load (*p* = 0.005)	Greater	14	8.0%	14	3.2%	13	5.4%
	About the same	87	49.4%	187	42.8%	83	34.7%
	Somewhat less	54	30.7%	174	39.8%	112	46.9%
	Considerably less	21	11.9%	62	14.2%	31	13.0%
Your ability to perform common procedures (e.g., physical exams, surgery, handling animals) (*p* = 0.006)	Greater	13	7.4%	13	3.0%	10	4.2%
	About the same	119	67.6%	290	66.2%	145	60.4%
	Somewhat less	36	20.5%	119	27.2%	63	26.3%
	Considerably less	8	4.5%	16	3.7%	22	9.2%
Your memory (*p* = 0.635)	Greater	3	1.7%	6	1.4%	6	2.5%
	About the same	105	59.7%	274	62.3%	159	66.0%
	Somewhat less	62	35.2%	151	34.3%	71	29.5%
	Considerably less	6	3.4%	9	2.0%	5	2.1%
Your ability to manage complicated clinical problems (*p* = 0.016)	Greater	29	16.4%	38	8.7%	15	6.3%
	About the same	114	64.4%	314	72.0%	166	69.2%
	Somewhat less	33	18.6%	79	18.1%	55	22.9%
	Considerably less	1	0.6%	5	1.1%	4	1.7%
Your ability to incorporate new modalities of diagnosis and treatment into your practice (*p* = 0.054)	Greater	32	18.3%	47	10.7%	24	10.0%
	About the same	107	61.1%	287	65.5%	157	65.4%
	Somewhat less	32	18.3%	94	21.5%	47	19.6%
	Considerably less	4	2.3%	10	2.3%	12	5.0%
Your ability to handle the stresses of veterinary medicine (*p* < 0.001)	Greater	39	22.0%	41	9.3%	22	9.1%
	About the same	55	31.1%	174	39.5%	123	50.8%
	Somewhat less	52	29.4%	155	35.2%	74	30.6%
	Considerably less	31	17.5%	70	15.9%	23	9.5%
Your ability to deal with difficult personalities at work (*p* = 0.080)	Greater	35	20.0%	75	17.2%	33	13.9%
	About the same	55	31.4%	169	38.8%	110	46.4%
	Somewhat less	53	30.3%	110	25.2%	60	25.3%
	Considerably less	32	18.3%	82	18.8%	34	14.3%
Your ability to empathize with clients (*p* < 0.001)	Greater	36	20.3%	61	13.9%	41	17.1%
	About the same	93	52.5%	269	61.4%	172	71.7%
	Somewhat less	36	20.3%	77	17.6%	25	10.4%
	Considerably less	12	6.8%	31	7.1%	2	0.8%
Your ability to recover from a long shift (*p* = 0.054)	Greater	13	7.5%	35	8.2%	6	2.7%
	About the same	53	30.5%	109	25.6%	77	34.7%
	Somewhat less	65	37.4%	172	40.5%	90	40.5%
	Considerably less	43	24.7%	109	25.6%	49	22.1%
Your level of emotional exhaustion (*p* = 0.023)	Greater	68	39.1%	150	34.3%	61	25.6%
	About the same	65	37.4%	194	44.4%	127	53.4%
	Somewhat less	20	11.5%	57	13.0%	31	13.0%
	Considerably less	21	12.1%	36	8.2%	19	8.0%
Your ability to recover from a night shift (*p* = 0.331)	Greater	3	5.0%	6	5.4%	3	4.3%
	About the same	7	11.7%	22	19.8%	17	24.6%
	Somewhat less	15	25.0%	23	20.7%	21	30.4%
	Considerably less	35	58.3%	60	54.1%	28	40.6%

### Retirement

**Figure 1:**
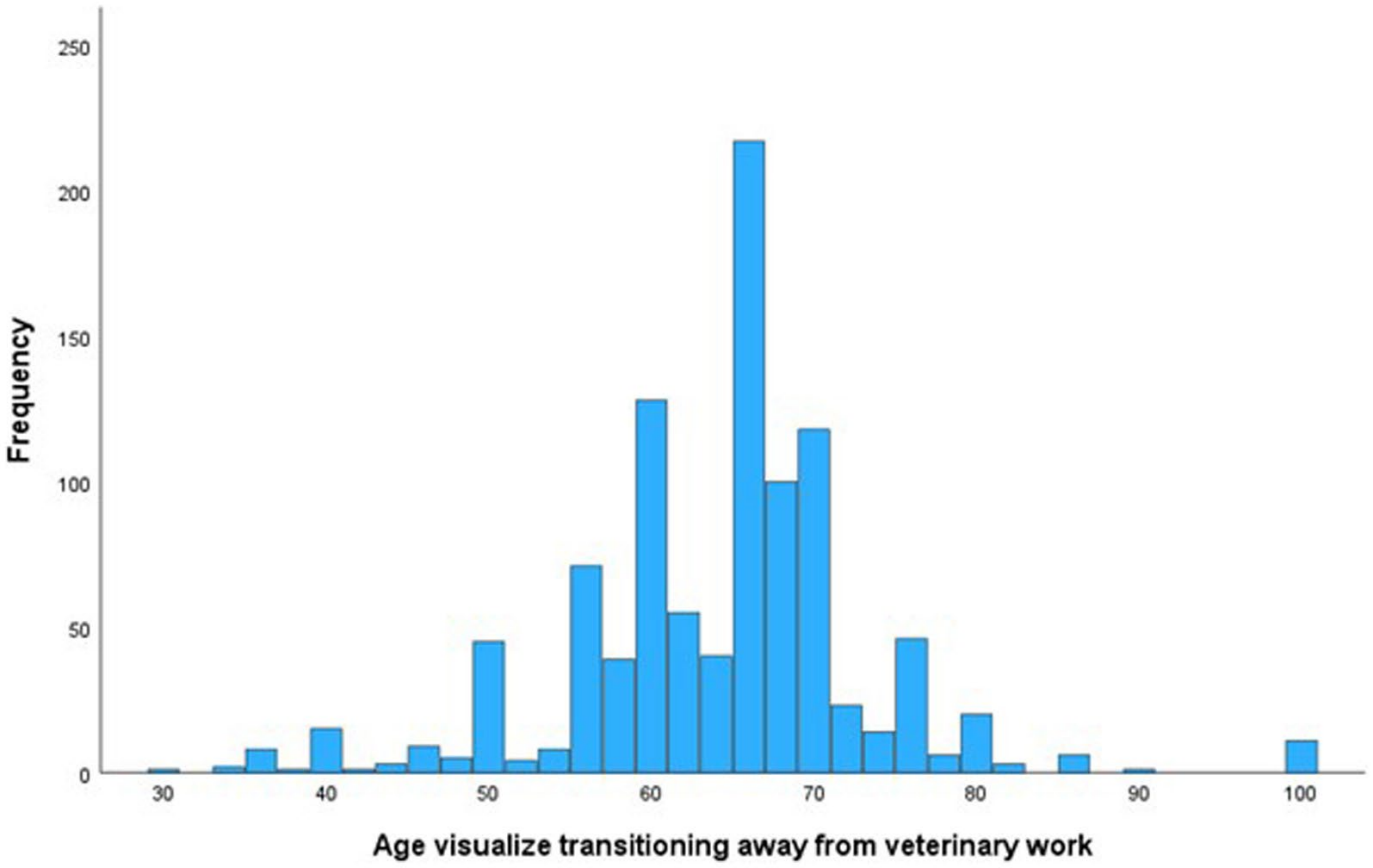
Reported age participants’ visualize transitioning away from veterinary work.

The mean age at which participants visualized transitioning away entirely (either to another type of non-veterinary work or retirement) from clinical veterinary work was 64 (SD = 9.34, median = 65). However, 23.4% reported an intended retirement age of >70 years, and several reported not ever intending to retire (Figure 1). When participants ages 45 and older were asked indicate their agreement to the statement ‘my current financial preparations for retirement are adequate’, most either agreed (298/913, 32.6%) or strongly agreed (324/913, 35.5%). Males were more likely to report agreeing that their current financial preparations for retirement are adequate (213/277, 76.9%) compared to females (408/633, 64.5%) (*p* < 0.001).

Participants were asked to indicate their thoughts about several possible concerns related to retirement. Almost half (426/912, 46.7%) reported concern about the loss of professional identity, and a third (311/912, 34.1%) reported concern about reduced social connections. Fewer (255/913, 27.9%) reported concern about how they would fill their time, or 121/708 (17.1%) about changes in their relationship with their partner/spouse. Females were more likely to report being concerned about the loss of professional identity (314/633, 49.6%) compared to males (111/276, 40.2%) (*p* = 0.033). No other gender differences were found (social connections: *p* = 0.220; fill time: *p* = 0.132; relationship with partner/spouse: *p* = 0.787).

Participants were asked where they access retirement information and guidance. Two thirds of participants reported using a personal financial advisor (705/1,055, 66.8%), a third used friends/family (367/1,055, 34.8%). Few used VIN (138/1,055, 13.1%) or AARP (national aging organization) resources (113/1,055, 10.7%). A fifth of participants (210/1,055, 19.9%) reported not seeking retirement guidance. Participants aged 45–54 (79/326, 24.2%) and 65 and older (54/256, 21.1%) were more likely to report currently seeking retirement information than participants ages 55–64 (77/473, 16.3%) (*p* = 0.019). Almost a third of respondents (339/1,052, 32.2%) reported that they did not have adequate information about retirement. Males were more likely to report feeling that they have adequate information about retirement (231/307, 75.2%) compared to females (481/743, 64.7%) (*p* < 0.001). Participants were asked to share what additional retirement resources would be useful. Response themes included financial planning and investment advice, understanding retirement accounts (401k, IRAs, social security), estimating retirement expenses and income needs, retirement calculators and checklists, information on healthcare options (Medicare, insurance), guidance on selling/closing a veterinary practice, and connecting with other retired veterinarians for advice and experiences.

## Discussion

Our study focused on factors surrounding veterinarians’ decisions to reduce their clinical hours or retire. Although substantial attention has been directed toward veterinarian numbers and supply, there has been relatively little research examining retirement or pre-retirement clinical activity. The participants in our study visualized transitioning away from veterinary work at a mean age of 64, similar to the average retirement age of workers in the US (1). When asked if, and how, they plan to change their amount of clinical work over the next 5 years, 61% reported they plan to decrease their clinical work, and 31% reported they plan to stop veterinary work entirely. Notably, 35% of veterinarians ≤44 years of age reported they plan to decrease their clinical hours and 7% indicated they plan to stop entirely. These figures reflect other studies suggesting that 29–49% of health care professionals, and 27% of veterinarians, would like to reduce their work hours or work part-time (20, 30).

While it is typical, and often desired, for medical providers to reduce their activity level in the years immediately preceding retirement (31), our study suggests that the desire to reduce one’s clinical work is felt by over a third of younger veterinarians. A reduction in workload can impact the perceived number of currently practicing veterinarians and lead to overestimating the number of active veterinarians (31).

When the participants who indicated they plan to stop/reduce the amount of their clinical work over the next 5 years were asked to identify the reasons for their decision, the most common reasons were to have more free time for oneself and/or family/friends, maintain good health, and because of burnout. When assessing predictors of the decision to stop/reduce the amount of their clinical work over the next 5 years, we found that, after age, burnout was the largest predictor. Additional predictors included job fulfillment, debt load, children, and relationship status. Those more likely to indicate they plan to stop/reduce the amount of their clinical work over the next 5 years had high levels of burnout, low levels of job fulfillment, and were married/partnered with no children and no outstanding high debt.

These results support earlier work by Nolen who found the main reasons for early retirement relate to work-life balance and mental health (32). Instead of being separate entities, burnout and health are intertwined, with burnout identified as a known risk factor for poorer physical health, impaired regulation of thought, action, and emotion, increased risk for early onset of age-related diseases, and premature death (33). Burnout has been identified as a predictor in physician’s decisions to leave a practice (34). In our study, we found that 34% of participants scored above the cut off for burnout, with younger veterinarians reporting higher levels of burnout than older veterinarians.

Burnout, characterized as a syndrome resulting from chronic workplace stress, is prominent in veterinary medicine (5, 35, 36), with veterinarian burnout scores found to be approximately 40% higher than physicians (37). Women veterinarians, associates, and those with less experience, exhibit higher burnout rates than men, practice owners, or those with more experience (36, 38, 39).

The economic impacts of burnout are significant and often measured through turnover and a reduction in hours (40). A reduction in working hours, or even the intention to reduce one’s hours because of burnout, can lead to lost revenue due to an increase in medical errors and poorer patient outcomes (35, 40, 41). Much has been written about burnout, including the need to address it at an organizational level, versus an individual level (35). While individual self-care to combat burnout can be helpful, it has been suggested that hospitals carry the primary responsibility to reduce and prevent burnout, including the provision of good organizational leadership (35, 42–46). Effective leadership can reduce burnout and stress and have a positive impact on burnout (35, 44). Facets of leadership that have been shown to positively impact burnout include keeping people informed, encouraging feedback from employees, and providing recognition of quality job performance (44). Future work on burnout should focus on organizational interventions that are tailored to each individual practice, but focus on quality leadership, improved workflow, and increased utilization of support staff (35). Veterinary organizations could play a key role in these efforts.

When participants who reported plans to stop/reduce the amount of their clinical work over the next 5 years were asked what might entice them to retain their current number of clinical hours, the most commonly endorsed factors included reduced workload or shorter hours, financial incentivization, and improved working conditions.

Studies suggest that the baby-boomer generation’s work ethic that mandates long days and little work life balance is not what current health providers endorse (14). Expectations for high weekly work hours can lead to conflicts between personal/professional responsibilities and are one of the greatest predictors of physician burnout, and intent to reduce clinical work hours or leave the field (47). Instead of the 80 h work week, the majority of current physicians prefer to work part-time, with younger physicians in particular seeing part time work as the way to meet their work life balance needs and decrease burnout (30). In our study, 60% of respondents noted that they feel they work too much, sentiments reflected in recent veterinarian studies (14, 48).

Previous research suggests that interventions which focus on reducing job demands and the number of working hours can improve health providers’ well-being and help with retention (30). In addition to a reduction in overall hours, this could also entail a shifting of duties to include more non-clinical duties such as teaching and mentorship, thereby helping both those feeling burned out as well as younger professionals.

Related to future career plans, we asked participants ≥50 years of age to compare their current status with that of 5 years ago on several job-related factors. The factors noted by the largest number of participants to have declined include the ability to recover from a night shift or a long shift, and the ability to deal with difficult personalities at work. Although participants ≥65 were more likely to report declined abilities in several areas, it is important to note that participants 50–54 years of age reported decreased abilities in several factors critical to clinical work. For example, 39% of participants 50–54 years of age noted a decreased ability to practice veterinary medicine the way they want based on their physical health, 43% noted deceased ability to manage a heavy patient load, 25% noted a decrease in the ability to perform common procedures, and 47% noted a decrease in the ability to handle the stresses of veterinary medicine. While, to our knowledge, no studies have reported self-perceived cognitive and physical age-related declined abilities among veterinarians, physician studies have found that they are able to self-identify cognitive or physical declines as they age, and take these factors into consideration when deciding when to retire (19). Future research exploring the impact of veterinarians’ perception of their cognitive and physical declines on decisions to reduce or stop clinical work is needed. Results from our study suggest that intervention and support for practicing veterinarians to help them stay in the field is needed well before typical retirement age. Caution should be taken that this information be used to help support veterinarians rather than, even inadvertently, discriminate when hiring or reviewing performance. Competing with a desire to retire are concerns about associated potential negative changes, including loss of identity or social connections, how one structures one’s time, and changes in one’s relationship with partner/spouse. We found that nearly half of our participants reported feeling concerned about the loss of professional identity and a third reported concern about reduced social connections or how they would fill their time. Supporting veterinarians as they retire is critically important, not only with regards to finances and logistics, but also with the associated personal and emotional aspects involved. It would be useful for veterinary associations to take on this effort by providing webinars, trainings, and a system to help connect people contemplating retirement with retired veterinarians for advice and guidance. This could not only help support those leaving the field but could help those already retired remain active and give back to younger generations.

There are several limitations to our study. Our sample consisted of a small percentage of VIN members, so caution is suggested when generalizing to other veterinarians since we cannot be certain that our sample represents the overall veterinary population. Moreover, because VIN membership is a paid subscription, the financial status of our sample might not represent the overall veterinary population. Potential response bias is another limitation. It is possible that individuals who have been thinking about reducing their hours or retiring may have been more likely to respond. Additionally, some participants might have been more likely to indicate they plan to decrease or stop clinical veterinary work for administrative work if a more detailed explanation or examples of veterinary related administrative work was provided. Yet, the percentages of participants who reported wanting to reduce their hours or retire resemble other national health provider surveys (30, 38).

In conclusion, our study found a significant number of veterinarians hope to reduce or stop clinical work within the next 5 years, often citing burnout as a factor in their decision. With the current shortage of veterinarians, plans by a significant number of practitioners to decrease their hours or leave the field will likely only exacerbate the problem by increasing the workload of others and increasing burnout rates. However, we also found that many of those contemplating a reduction in hours or retirement could be incentivized to stay if properly motivated. It is critically important, not only those contemplating leaving the field, but the colleagues they will leave behind, to proactively work with these individuals to address their needs through systemic organizational changes and personal support. For those who do make the decision to retire, it is suggested that we repay them for their service by providing support to ensure a successful transition and opportunities to stay involved in the field.

## Data availability statement

The raw data supporting the conclusions of this article will be made available by the authors, without undue reservation.

## Ethics statement

The studies involving humans were approved by the Internal Review Board – Colorado State University. The studies were conducted in accordance with the local legislation and institutional requirements. The Ethics Committee/Institutional Review Board waived the requirement of written informed consent for participation from the participants or the participants’ legal guardians/next of kin because the study was determined to be exempt by the Internal Review Board of Colorado State University.

## Author contributions

LK: Conceptualization, Formal analysis, Investigation, Methodology, Writing – original draft, Writing – review & editing. MR: Conceptualization, Data curation, Formal analysis, Methodology, Writing – review & editing.
