# Supplementary material for: Career transition plans of veterinarians in clinical practice
Source: Front Vet Sci. 2024 Jul 26;11:1433891. doi: 10.3389/fvets.2024.1433891 (PMC11310143; doi:10.3389/fvets.2024.1433891)
Supplement: Supplementary file 1 [file Data_Sheet_1.PDF]

Veterinarians and retirement plans

We are interested in how you feel about transitioning away from veterinary medicine. If you are a practicing veterinarian, we are interested in your views.

My name is Lori Kogan and I am a researcher from Colorado State University in the Clinical Sciences department and Principal Investigator of this study. We are conducting a research study to learn more about how veterinarians feel about transitioning away from veterinary medicine. The title of the study is “Veterinarians and retirement plans”.

If you are a practicing veterinarian, we would like to ask you to take this anonymous online survey. Participation will take approximately 5-10 minutes. Your participation in this research is voluntary. If you decide to participate in the study, you may withdraw your consent and stop participation at any time without penalty.

We will not collect your name or personal identifiers. When we report and share the data to others, we will combine the data from all participants. This data will be used for research purposes. While there are no direct benefits to you, we hope to gain more knowledge on how veterinarians feel about their work.

It is not possible to identify all potential risks in research procedures, but the researcher(s) have taken reasonable safeguards to minimize any known and potential (but unknown) risks. If you have any questions about the research, please contact Lori Kogan at lori.kogan@colostate.edu. If you have any questions about your rights as a volunteer in this research, contact the CSU IRB at: CSU_IRB@colostate.edu; 970-491-1553.

---

1) Are you any of the following?*

( ) Retired

( ) Unemployed

( ) Intern

( ) Resident

( ) None of the above

Demographics

2) How old are you?*

____

3) What is your gender?

( ) Female

( ) Male

( ) Non-binary

( ) Prefer not to answer

4) What best describes your relationship status?

( ) Partnered or married

( ) Single

5) How many children under the age of 18 are you responsible for?

( ) None

( ) One

( ) Two

( ) Three

( ) Four

( ) Five

( ) Six

( ) Seven

( ) Eight or more

6) Are you responsible for the care and/or financial upkeep of elderly or disabled family members?

( ) Yes

( ) No

7) Do you have any outstanding large debts (e.g. student loans, mortgage, business loans, etc)

( ) Yes

( ) No

Work situation

8) How many hours do you typically work per week (include all paid work you perform – veterinary and non-veterinary)?
Estimate the average to the nearest hour.*

____

9) Which of the following types of paid work do you perform at least weekly?
Select all that apply*

[ ] Clinical (i.e., working directly with animals)

[ ] Non-clinical veterinary-related (i.e., practice management duties, administrative duties, teaching, consulting, industry, research)

[ ] Non-veterinary (e.g. property management etc)

10) What proportion of your weekly paid work do you spend in each of these categories?

________Option 1

________Option 2

Clinical Work

11) What proportion of your clinical work do you spend with each of the following animal types?

________Small animal (dogs, cats)

________Large animal (cattle, sheep, goats, pigs etc)

________Equine

________Exotic animals (e.g. pocket pets, fish, avian, poultry etc)

________Wildlife (native or wild fauna)

12) What best describes your clinical work situation?

( ) Brick-and-mortar private practice

( ) Brick-and-mortar corporate practice

( ) Mobile

( ) Relief

( ) Academic

( ) Shelter

( ) Other - Write In (Required): ____*

Non-Clinical Work

13) What proportion of your non-clinical veterinary-related work do you spend in each category?
If you select "Other", please describe in the Comments below the question.

________Practice management or administration

________Book-keeping

________Teaching

________Consulting

________Industry

________Research

________Other

Comments:

Non-Veterinary work

14) What non-veterinary work do you perform?
Please describe

____

____

____

____

Current mental wellbeing

15) Which statement best reflects your current work/life balance?

( ) I feel I work way too much, leaving little to no time for home/personal life

( ) I feel I work somewhat too much, leaving some, but not enough, home/personal life

( ) I feel I work the right amount, leaving me enough time for home/personal life

( ) I feel I would like to work a bit more, I have plenty of time for home/personal life

( ) I feel I would like to work a great deal more, I have too much time for home/personal life

16) Rate your current level of job satisfaction with clinical veterinary work

0 __________________________[__]_______________________________ 10

17) Rate your current level of job satisfaction with non-clinical veterinary-related work

0 __________________________[__]_______________________________ 10

18) Rate your current level of job satisfaction with non-veterinary work

0 __________________________[__]_______________________________ 10

19) In the past two weeks, how true are the following statements about your relationship to clinical veterinary work?

	**Not true at all**	**Somewhat true**	**Moderately true**	**Very/quite true**	**Extremely true**
I feel happy at work	( )	( )	( )	( )	( )

I feel worthwhile at work	( )	( )	( )	( )	( )
My work is satisfying to me	( )	( )	( )	( )	( )
I feel in control when dealing with difficult problems at work	( )	( )	( )	( )	( )
My work is meaningful to me	( )	( )	( )	( )	( )
I am contributing professionally (e.g. patient care, teaching etc)	( )	( )	( )	( )	( )

20) During the past two weeks doing clinical veterinary-related work I have felt…

	**Not at all**	**A little**	**Moderately**	**A lot**	**Extremely**
A sense of dread when I think about work I have to do	( )	( )	( )	( )	( )
Physically exhausted at work	( )	( )	( )	( )	( )

Lacking in enthusiasm at work	( )	( )	( )	( )	( )
Emotionally exhausted at work	( )	( )	( )	( )	( )

21) During the past two weeks my clinical veterinary-related work has contributed to me feeling...

	**Not at all**	**A little**	**Moderately**	**A lot**	**Extremely**	**Not applicable**
Less empathetic with my patients	( )	( )	( )	( )	( )	( )
Less empathetic with my colleagues	( )	( )	( )	( )	( )	( )
Less sensitive to others' feelings/emotions	( )	( )	( )	( )	( )	( )
Less interested in talking with clients	( )	( )	( )	( )	( )	( )
Less connected with my patients	( )	( )	( )	( )	( )	( )
Less connected with my colleagues	( )	( )	( )	( )	( )	( )

Transitioning

22) Within the next 5 years, do you plan to change the amount or type of paid work you do?*

( ) Yes

( ) No

23) How do you plan to change the amount or type of paid work you do?*

	**Increase**	**Leave unchanged**	**Decrease**	**Stop entirely**	**Not applicable**
Clinical veterinary work	( )	( )	( )	( )	( )
Non-clinical veterinary work	( )	( )	( )	( )	( )
Non-veterinary work	( )	( )	( )	( )	( )

Reducing clinical hours

24) Please indicate the reasons you are considering reducing or ceasing your clinical hours (select all that apply)

[ ] Declining reimbursement for clinical care
[ ] Financial security/insufficient financial incentive to stay
[ ] Feeling burned out
[ ] To have more free time for self +/- family/friends
[ ] To maintain good health
[ ] Personal health problems
[ ] Family members' health problems
[ ] To pursue veterinary-related administrative/leadership opportunities
[ ] To pursue veterinary-related research or medical education opportunities
[ ] To pursue non-veterinary business or employment ventures
[ ] Pressure of work
[ ] Work schedule (overnights, on call, etc.)
[ ] Reduced job satisfaction
[ ] Retirement of spouse/partner
[ ] Possibility of deteriorating cognitive skills needed to perform clinical work
[ ] Possibility of deteriorating physical ability needed to perform clinical work
[ ] Other - Write In (Required): ____*

(untitled)

How important are each of these reasons in your desire to reduce or cease clinical work within the next 5 years? 1 star = not very important; 5 stars = extremely important.

	Importance

Enticements to maintain clinical hours

25) Which, if any, of the following factors would entice you to maintain your current number of clinical hours?

[ ] Work-load reduction/shorter hours

[ ] Reduction of on-call or emergency commitments

[ ] Reduction of work-related bureaucracy

[ ] Financial incentivization

[ ] Improved working conditions, other than hours

[ ] Development opportunities

[ ] More involvement in direct patient care

[ ] Less involvement in direct patient care

[ ] Other - Write In (Required): ____*

[ ] None of these

Reducing non-clinical hours

26) Please indicate the reasons you are considering reducing or ceasing your non-clinical veterinary-related hours (select all that apply)

[ ] Declining reimbursement for non-clinical care

[ ] Financial security/insufficient financial incentive to stay

[ ] Feeling burned out

[ ] To have more free time for self +/- family/friends

[ ] To maintain good health

[ ] Personal health problems

[ ] Family members' health problems

[ ] To pursue veterinary-related administrative/leadership opportunities

[ ] To pursue veterinary-related research or medical education opportunities

[ ] To pursue non-veterinary business or employment ventures

[ ] Pressure of work

[ ] Work schedule (overnights, on call, etc.)

[ ] Reduced job satisfaction

[ ] Retirement of spouse/partner

[ ] Possibility of deteriorating cognitive skills needed to perform non-clinical work

[ ] Possibility of deteriorating physical ability needed to perform non-clinical work

[ ] Other - Write In (Required): ____*

(untitled)

How important are each of these reasons in your desire to reduce or cease non-clinical veterinary-related work within the next 5 years? 1 star = not very important; 5 stars = extremely important.

	**Importance**

Enticements to maintain non-clinical hours

27) Which, if any, of the following factors would entice you to maintain your current number of non-clinical veterinary-related hours?

[ ] Work-load reduction/shorter hours

[ ] Reduction of on-call or emergency commitments

[ ] Reduction of work-related bureaucracy

[ ] Financial incentivization

[ ] Improved working conditions, other than hours

[ ] Development opportunities

[ ] More time for non-clinical work by having less involvement in direct patient care

[ ] Other - Write In (Required): ____*

[ ] None of these

Transitioning away from veterinary medicine

28) We understand that things can always change, but right now, at what age do you visualize transitioning away entirely (either to another type of work or retirement) from veterinary work?*

If you have not thought about this at all, please note this in the comment box

____

Comments:

Approaching Retirement

29) Please indicate your level of agreement with the following statements

	Strongly agree	Agree	Neutral	Disagree	Strongly disagree
My current financial preparations for retirement are adequate	( )	( )	( )	( )	( )

I have concerns about the loss of my professional identity when I retire	( )	( )	( )	( )	( )
I have concerns about having reduced social connections when I retire	( )	( )	( )	( )	( )
I have concerns about how I will fill my time when I retire	( )	( )	( )	( )	( )
I have concerns about changes in my relationship with my spouse/partner when I retire	( )	( )	( )	( )	( )

30) In general, how would you rate your physical health?

( ) Excellent

( ) Very good

( ) Good

( ) Fair

( ) Poor

31) For the following questions, please compare your current status with that of five years ago

	Greater	About the same	Somewhat less	Considerably less	Not applicable
Your ability to practice veterinary medicine the way you want based on your physical health	( )	( )	( )	( )	( )
Your ability to practice veterinary medicine the way you want based on your cognitive abilities	( )	( )	( )	( )	( )
Your ability to manage a heavy patient load	( )	( )	( )	( )	( )
Your ability to perform common procedures (e.g. physical exams, surgery, handling animals)	( )	( )	( )	( )	( )

Your memory	( )	( )	( )	( )	( )
Your ability to manage complicated clinical problems	( )	( )	( )	( )	( )
Your ability to incorporate new modalities of diagnosis and treatment into your practice	( )	( )	( )	( )	( )
Your ability to handle the stresses of veterinary medicine	( )	( )	( )	( )	( )
Your ability to deal with difficult personalities at work	( )	( )	( )	( )	( )
Your ability to empathize with clients	( )	( )	( )	( )	( )
Your level of emotional exhaustion at the end of a shift	( )	( )	( )	( )	( )

Your ability to recover from a long shift	( )	( )	( )	( )	( )
Your ability to recover from a night shift	( )	( )	( )	( )	( )

Retirement resources

32) Where do you go for retirement information/guidance? (select all that apply)

[ ] AVMA or national veterinary association

[ ] VIN

[ ] DVM360 or other trade magazines

[ ] AARP or national ageing association

[ ] Personal financial advisor

[ ] Family or friends

[ ] Other - Write In (Required): ____*

[ ] I don't currently seek retirement information or guidance

33) Do you feel you have adequate or sufficient information about retirement?*

( ) Yes

( ) No

34) What additional resources would be helpful?

____

Thank You!
